# Oncolytic Adenoviruses: Strategies for Improved Targeting and Specificity

**DOI:** 10.3390/cancers12061504

**Published:** 2020-06-09

**Authors:** Praveensingh B. Hajeri, Nikita S. Sharma, Masato Yamamoto

**Affiliations:** 1 Division of Basic and Translational Research, Department of Surgery, University of Minnesota, MMC 195, 420 Delaware St SE, Minneapolis, MN 55455, USA; phajeri@umn.edu (P.B.H.); sharm249@umn.edu (N.S.S.); 2 Masonic Cancer Center, University of Minnesota, Minneapolis, MN 55455, USA; 3 Stem Cell Institute, University of Minnesota, Minneapolis, MN 55455, USA

**Keywords:** adenovirus vector, oncolytic adenovirus, library, targeting, promoter, screening

## Abstract

Cancer is a major health problem. Most of the treatments exhibit systemic toxicity, as they are not targeted or specific to cancerous cells and tumors. Adenoviruses are very promising gene delivery vectors and have immense potential to deliver targeted therapy. Here, we review a wide range of strategies that have been tried, tested, and demonstrated to enhance the specificity of oncolytic viruses towards specific cancer cells. A combination of these strategies and other conventional therapies may be more effective than any of those strategies alone.

## 1. Introduction

Cancer is a major health problem, posing a significant burden to individual patients and to society [1,2] (https://gco.iarc.fr/). Large efforts have been made to understand its causes and mechanisms of disease progression. Although several advanced therapeutic options based on them are now available, only a few cancer types can be treated effectively if curative surgical resection is not possible [3]. In the vast majority of cases, improving the quality of life of patients even slightly is a practical and significant goal to achieve. Among the available treatments, most of them unfortunately lack cancer specificity, leading to a range of systemic adverse effects that diminish a patient’s quality of life, which is still a big issue [3,4]. To improve patient outcomes, researchers have been focused on the development of more cancer-specific, targeted therapies [3,5,6,7,8]. 

In general, current strategies of drug development aim to modify the function of a target protein in order to slow down tumor growth or possibly decrease tumor volume. This strategy requires targets to be differentially expressed in tumors, and also functionally important for tumorigenesis and progression [6,9,10,11]. Numerous high-throughput genomic and proteomic studies comparing healthy and cancerous cells have identified many such potential drug targets [9,12,13]. These putative targets are then subjected to high-throughput screening with libraries of potential drug candidates, such as peptides, antibodies, natural compounds, chemicals, and aptamers [10,14,15,16,17,18,19,20,21,22]. Selected molecules that specifically bind to the target are considered for further functional validation [17]. Unfortunately, many potentially druggable genes were found to be difficult to target by this method. Most of these screening experiments showed that despite specific binding of small molecules to tumor targets, the inhibitory or modifying effects of a large fraction of molecules were insufficient to alter their functions and may also exhibit significant toxicity [17,23,24]. Without strong inhibitory or modifying effects, these molecules cannot be developed for therapy under conventionally with methods [24,25,26]. Such issues have led to a lack of successful drug candidates [3,9,10,17]. 

In such situations, tumor targeting by viruses provides an excellent alternative. The natural ability of viruses to interact with cell surface proteins to gain entry into cells makes them attractive tools for targeted therapy [27,28]. If a virus can be engineered to interact with specific proteins or receptors in a cancerous cell, it can enter the cell to deliver therapeutic cargo or kill the cell by infection inducing cytolysis [27,29,30]. A major advantage of viruses over small molecules is that the target protein need not be functionally important to the tumor biology. Instead, it must only be specifically expressed or significantly overexpressed in a target cell [29,30]. Therefore, any gene unique to tumors, irrespective of its functional importance, can be subject to targeting. This dissociation of gene expression from functional relevance eliminates a major limitation, bringing hundreds of genes previously deemed undruggable back into the pool of potential therapeutic targets. This significantly improves the chances of identifying and developing new targeted therapies.

Many viruses cause lysis of infected cells at the end of their infection cycle. Among them, the viruses which are designed to kill cancerous cells are called oncolytic viruses (OVs) [27,28,31,32,33,34]. Many different viruses have been exploited for this purpose, most notably adenoviruses (AdV) [35], vesicular stomatitis virus (VSV) [36], herpes simplex virus (HSV) [37], vaccinia virus [38], reovirus [39,40], and Seneca valley virus [41,42]. Depending on the type of cancer, method of targeting, and therapeutic cargo to be delivered, some viruses may be more suitable than others. Here, we will focus on using adenoviruses as oncolytic viruses and discuss various strategies that have been employed and demonstrated to be effective in achieving a more specific targeting of cancer cells.

## 2. Adenoviruses as Vectors for Gene Therapy and Oncolytic Viruses

Adenoviruses are popular gene delivery vectors [43]. They can effectively infect both dividing and non-dividing cells [44]. Their double-stranded DNA genome remains episomal, rarely integrating into the host genome [45]. Additionally, while adenoviruses are very common pathogens to humans, they usually cause only mild symptoms in the upper airway, liver, urinary tract, tonsils, enteric, renal, and ocular tissues [12].

Adenoviruses are a family of icosahedral, non-enveloped viruses. Based on serology and genomic sequences, AdVs have been grouped into seven species, each including several types/subtypes [46]. Their capsid is comprised of four structural proteins (hexon, penton, fiber, and pIX), each of which contributes to interaction with the host cell surface. Depending on the type of virus, they can bind to various cell surface proteins to facilitate entry into target cells [47,48,49]. The knob region of fiber can bind to Coxsackievirus and adenovirus receptor (CAR), Vascular cell adhesion molecule-1 (VCAM), CD80, CD86, MHC1, and Scavenger receptors (SRs) [47]. The shaft of fiber can bind to heparan sulphate proteoglycans (HSPGs) [48] and RGD (Arginine(R)-Glycine(G)-Aspartate(D)) motif in the penton can bind to integrins, CD46, and sialic acid (SA) [47]. In some cases, adenoviral protein interactions can be indirect, mediated by a bridging molecule. Hexon can bind to coagulation factor X (FX) [49], which in turn helps in binding to HSPG. Different serotypes show wide ranges of affinities for these binding partners. There is some discrepancy in determining the most important receptor in a particular cell type, however in most cases several receptors play significant roles. Understanding interactions and affinities between viral proteins and host receptors has helped in designing improved strategies of virus detargeting (abolishing virus affinity towards natural targets) and retargeting (generating affinity towards newer proteins or domains).

AdVs are popular as gene therapy vectors and are the subject of over 100 clinical trials [46]. Among them, Ad2 and Ad5 are the most widely used and widely studied for gene therapy. However, there are some limitations to their use. (1) A significant number of people possess antibodies against common adenoviruses, making it difficult for conventional AdVs to be used for systemic injection. However, in recent years, this thought process has changed and immune response has been somewhat exploited to favor AdV-mediated therapy [50]. (2) AdVs are quickly sequestered to organs such as the liver and lungs after systemic injection. Residential macrophages (e.g., Kupffer cells) play an important role in this process. In one extreme case, high-dose intravascular injection induced a fatal cytokine storm [51]. Erythrocytes and platelets also contribute to sequestration of viral particles upon intravenous delivery [52,53,54]. In spite of these limitations, an increasing understanding of adenovirus biology has led to designing novel strategies to overcome such limitations, and significant progress has been made.

Detargeting AdVs away from their natural interactions and retargeting of AdVs towards a specific target in an intended cell are very crucial processes in developing a targeted therapy for cancer. Here, we will discuss various strategies employed to achieve detargeting, retargeting, and delivery of novel therapeutic molecules with a few selected examples.

## 3. Strategies for Specific Targeting of AdV

Broadly speaking, detargeting and specific retargeting can be achieved in multiple ways: (Section 3.1) by selective binding or retargeting towards proteins uniquely expressed or overexpressed on the surface of specific cells; (Section 3.2) by selective expression of effectors (inhibitors or enhancers) in specific cells; (Section 3.3) by inducing conditional or selective replication of viruses in specific cells; (Section 3.4) by combining these methods.

### 3.1. Selective Retargeting Towards Proteins Uniquely Expressed or Overexpressed on the Surface of Specific Cells

Based on structural and functional studies, we have a decent understanding of how viral proteins interact with host receptors. By altering the sequences of key interacting amino acid residues and domains, their affinity towards natural receptors can be abolished (Section 3.1.1, detargeting) and redirected towards a new protein (Section 3.1.3, retargeting). Since it is difficult to predict how sequence changes may alter the affinity towards an intended target, retargeting towards a specific target is a very challenging process. High-throughput screening to look for such sequences may be the best way (Section 3.1.2, infectivity selective screening for specific targeting) to find retargeting sequences and molecules [55]. Most structural proteins, including hexon, pIX, fiber, and penton, have been exploited by these approaches.

#### 3.1.1. Detargeting of AdV Structural Proteins

##### Detargeting of Hexon and pIX

Hexon is the most abundant (240 trimers) structural protein in the AdV capsid and is also the main cause of AdV sequestration to the liver [56,57]. Hexon binds to factor-X (FX), a soluble coagulation factor found in blood plasma, facilitating viral entry into Kupffer cells via scavenger receptors (SR) [58,59]. Abolishing this interaction will greatly reduce sequestration, allowing circulating AdV to be distributed to other tissues. This can be accomplished by several mechanisms, including by introducing mutations into the hyper variable region (HVR5) of hexon [60], inserting heterologous sequences, swapping the whole HVR [57,59,61], and by using drugs (such as warfarin and snake venom protein, factor X-binding protein (X-Bp) [62,63].

Some naturally occurring amino acid sequences (peptides) with known binding characteristics have been used for detargeting and retargeting. A well-known example is the RGD motif found in penton in AdVs [64,65]. This peptide is known to interact with integrins that have been slightly modified into RGD-4C and incorporated into HVRs of hexon and the HI-loop of fiber [64,65,66,67,68,69]. In combination with other modifications, this could efficiently detarget fiber from CAR and increase its affinity towards integrin enriched cells. Some studies have shown promising results and progression through clinical trials [70,71,72,73,74,75]. However, we think looking for naturally occurring motifs and incorporating them into AdV structural proteins to see if they retain their affinity and specificity offers limited options for newly discovered targets of interest.

Another strategy utilizes information from peptide-based screenings that have been performed over the past few decades on key cancer-related drug targets. Such screenings are mostly done by using phage display and/or chemically synthesized libraries. Several potential candidates that specifically bind to individual drug targets have been identified. These peptides, irrespective of their function, can be incorporated into hexon to retarget oncolytic adenoviruses (OAdV). A few successful examples have been described [64,65,76,77,78]. Ghosh et al. introduced two such peptides into the HVR5 region of hexon to detarget it away from FX and retarget it towards skeletal muscle cells, which otherwise are not susceptible to AdV binding [78,79]. It seems that small peptides may be a better choice than larger protein domains, as they may not affect the structural integrity and assembly of the virus [80].

In comparison, altering sequences of hexons seems to be a much safer option than pIX, keeping the structural integrity of capsids in mind. Since hexons and pIX exist in high copy numbers within the virus capsid, a slight increase in their affinity towards an intended target may lead to a stronger cumulative effect on binding [81,82,83].

##### Detargeting of Fiber

Fiber is another major AdV structural protein that interacts with host receptors. Fiber consists of two components: (1) the shaft, projecting outward from each apex of the capsid structure; and (2) the knob, located at the distal end of the shaft. The knob structure is stabilized by multiple loops. Structural and mutagenesis studies have shown that the AB-loop of fiber is critical for interaction with CAR, while the c-terminal domain and HI-loop were not involved [84]. Therefore, the HI-loop and C-terminal domain can be manipulated without affecting the fiber’s ability to interact with CAR. Since they are positioned towards the terminus, additional amino acids introduced in these domains may remain out of the core structure of knob, and therefore may not affect its structural stability.

As with hexons, peptides selected by high-throughput screening methods, as mentioned before, have been introduced into the HI-loop or C-terminus to detarget and retarget fiber in AdVs [66,67,85,86]. A large number of successfully retargeted AdVs towards neuronal cells, brain endothelial cells, vascular endothelial cells, prostate cancer, lung cancer, pancreatic cancer, muscle cells, and receptors-like epidermal growth factor receptor (EGFR) have been reported [66,67,85,86,87,88,89,90,91,92,93,94,95,96,97,98,99]. Similar to hexon-based studies, keeping the structural stability in mind, small peptides are widely used. However, proteins as large as 83 amino acids have been introduced into this domain, but the viral packaging was somewhat affected [87,88,98,99]. Therefore, smaller proteins and peptides seem to be the better options.

Simply modifying the HI-loop or c-terminus while keeping the AB-loop intact may not fully abolish fiber–CAR interactions, which is another major reason behind sequestration of AdVs. Hence, complete abolition of fiber–CAR interaction by modifying the AB-loop would be more beneficial. Miura et al. showed improvements in many screening experiments after modifying the AB-loop region [55]. Sato et al. used AB-loop-modified AdV libraries to screen for candidates that specifically bind to CD133. They demonstrated that a peptide sequence “TYMLSRN” introduced into the AB-loop efficiently retargeted the AdV towards CD133 and away from CAR [100]. Similarly, the “VTINRSA” peptide could retarget AdVs to mesothelin [55]. RGD motif and its derivatives were used to modify the fiber, and many such modified viruses are undergoing clinical trials [64,65,66,67,68,69,70,71,72,73,74,75].

As mentioned above, identifying an appropriate retargeting sequence to replace wild-type sequences is very challenging. A novel alternative to replacing native sequences with retargeting sequences was demonstrated by introducing a “universal acceptor domain” into structural proteins. A bridging molecule can then be used to facilitate the interaction between the virus and its target. The Fc region binding domain of Staphylococcus aureus protein A, biotin acceptor protein (BAP), and a FLAG^®^ peptide have been used as universal acceptor domains [101,102,103,104,105,106,107,108]. These “universal acceptor domains” are usually large proteins, and their incorporation into structural proteins may affect viral stability. Since the viral fiber protrudes outside the core capsid, we think it is a more suitable location for insertion of large protein domains than hexons which form the core of the capsid.

Swapping of fibers is also an option to change the affinity and evade immune response, at least temporarily [109,110,111,112,113,114]. The swapping of fibers and chimeric fibers has been very promising in early experiments and clinical trials, especially Ad5/3, Ad5/11, Ad5/9, and Ad5/35 [95,115,116,117,118,119], Since these fibers (Ad3 and Ad35 fibers) do not bind to CAR, detargeting from CAR becomes an inherent property. However, this may drive their affinity towards desmoglein, CD46, and other native receptors, leading to a different type of off-target effects. This can be overcome by combining this (swapping) with other strategies, such as adding a targeting peptide, to achieve effective detargeting and retargeting [64,68,112].

#### 3.1.2. Infectivity Selective Screening for Specific Targeting: Strategies and Importance

A typical novel drug discovery approach against a specific target mainly relies on screening of a large collection of small molecules and potential drug candidates. Several peptides, antibodies, single-chain fragment variables (ScFVs), nanobodies, and aptamers have been identified by high-throughput screening studies for their ability to bind specifically to a target [120]. Usually, such screenings are done in bacterial systems or in chemical mediums. One of the major concerns is that, the candidates selected under such alien environmental conditions may not retain their properties in eukaryotic cells or animal bodies, where they are ultimately intended to function. Further, they may not remain equally functional and effective when introduced into AdV structural proteins. In most cases, incorporation of such pre-identified motifs into AdV structural proteins affects the viral assembly due to structural deformation. Hence, vast majority of these candidates could not be used for retargeting of AdV [79,121].

**Figure 1:**
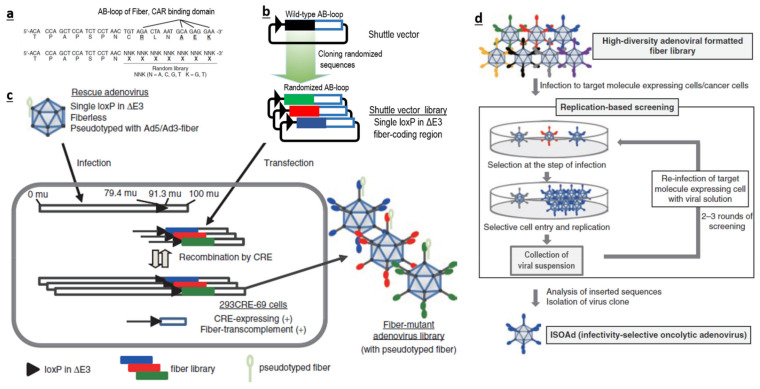
Method used to generate high-sequence diversity libraries and screening: the AB-loop region of the fiber (**a**) was replaced by various sequences, creating a library of shuttle vectors (**b**) and packaging this into functional replicating adenoviruses (**c**). Such libraries can be screened against a target of interest (**d**). Figure adapted from Miura, Y. et al. [55].

Therefore, if possible, it is more logical to avoid traditional bacterial or chemical screening methods. It could be better to directly generate a large-scale AdV library with huge sequence diversity at the desired loci (Figure 1a–c), and screen them for their ability to interact with a specific target (Figure 1d), instead of using a phage-based system in bacteria. Such AdV-based peptide display libraries can be created in the loops (like AB-loop, HI-loop) of fiber, c-terminal domains of fibers, HVRs of hexon and in pIX. We believe that AB-loop-based libraries are more suitable, as sequence manipulation in this region has a lower chance of affecting capsid assembly and can also abolish interactions between the wild-type AB-loop and CAR. This will consolidate both detargeting and retargeting within one library.

Generating libraries of eukaryotic viruses such as adenoviruses is a more complicated process than phage-based libraries or just plasmid-based libraries. Phage libraries are relatively easier to produce as they require only bacteria, which are more efficient than eukaryotic cells for DNA manipulation and transformation [122]. The difference in efficiency is huge and varies by several magnitudes. Hence, unfortunately, researchers had to choose between screening of a large number of candidates under alien conditions versus a limited number of candidates under native conditions [55,123]. Obviously, a library of a large number of candidates that can be screened under native conditions will be an ideal solution.

Several methods have been published, which describe the generation of large libraries, especially of phages and plasmids. Some reports have claimed to achieve libraries with a staggering diversity of over 40 billion unique candidates [124]. However, estimation of diversity in this report was based on statistical extrapolation of fewer than 100 sequences, rather than high-throughput next-generation sequencing (NGS) based methods [124]. Surprisingly, there are several other reports that have used NGS to sequence millions of candidates to more accurately determine the diversity of their libraries [125]. They unambiguously found that such high diversity (40 billion as claimed by others) did not exist in their libraries and it would be very difficult to achieve, even by scaling up their efforts. Very interestingly, in most of the published literature, we found that studies that claim huge diversity have not validated their claims with NGS, while those who used NGS never claim such large numbers. This correlation is revealing and it would be instructive to see the groups claiming huge diversity (billions) use NGS-based methods to validate their libraries and determine the actual diversity. Such studies are still awaited.

Our group has designed a novel approach for generating a library of AdVs with high sequence diversity in the AB-loop regions of fibers [55]. Screenings using this library has led to the identification of two successful candidates against mesothelin and CD133 [55,100]. Further, we have developed novel methods to generate an ultra-high sequence diversity library in the AB-loop region of fiber. The diversity has been validated to contain more than 100 million candidates by using stringent NGS-based methods [126]. This is, by far the largest, validated library (among plasmid, peptide, antibody, phage or recombinant virus based libraries) to our knowledge. Such a library with largest reported diversity which enables screening under native conditions will be very useful tool for screening for novel specificity determinants.

#### 3.1.3. Retargeting of AdV by Affinity Modifiers

##### Protein- and Peptide-Based Bridging Molecules

High-throughput screening of libraries and validation of selected candidates can be very resource intensive. Conceptually, a simpler way to overcome this, is by using “affinity modifiers” or bridging molecules. Typically, these are capable of binding to a viral particle on one end and to a potential target on the other end. Modifiers can be of a combination of a variety of specificity determinants, including peptide-based linkers, bispecific antibodies, diabodies, triabodies, antibody–ligand complexes, receptor–ligand complexes, and high-affinity binders to virus–ligand complexes.

The main purpose of these modifiers is to bring the virus in close proximity of an intended target cell and facilitate interaction. A few successful demonstrations are listed in Table 1 [127,128,129,130,131,132,133,134,135].

In general, due to the structural complexity of bridging molecules, their production, method of delivery, and stability pose limitations on their application. However, a large number of studies have demonstrated their effectiveness at least in vitro and when no other retargeting methods are available, these inconveniences may be overlooked. 

##### Soluble Receptors Conjugated to Ligands

Curiel’s group reported a novel way of designing bridging molecules by exploiting natural receptors of AdVs. Viruses have naturally evolved high affinity towards specific cell surface receptors. By using a soluble form of the same natural receptor, they could block (saturate) the native viral protein, leading to effective detargeting. This was demonstrated by using a soluble CAR (sCAR) conjugated to several ligands and ScFvs to retarget AdVs [136,137,138,139,140,141,142,143,144]. Hexon– Coagulation Factor (F)X (FX) binding was utilized in a similar manner by conjugating an ScFv against HER2/neu to the FX protein [145]. However, the in vivo efficiency of these methods has yet to be demonstrated.

Viruses, which express retargeting molecules as fused structural proteins (e.g., as nanobodies, peptides, or ScFVs fused to viral fiber, penton, pIX, or hexon) may not allow proper folding and post-translational modifications. This may render such retargeting molecules non-functional. This is somewhat expected, as the requirements of maturation of viral proteins and expressed targeting molecules are very different. Hence, expressing these targeting molecules separately and allowing them to undergo full maturation before assembling them into a functional virus–adapter complex could be more effective. However, expressing these retargeting molecules separately and then assembling them with virus particles into a functional complex before delivery has not been easy either. Synthetic leucine-zipper-based dimers [146,147] and bispecific T-cell engagers (BiTEs) are examples of retargeting molecules that may need extensive maturation. Several studies have tried to use them to increase tumor specificity, however the overall outcomes have not been significant [147,148,149,150,151,152,153,154,155].

### 3.2. Selective Expression of Effectors (Inhibitors or Enhancers) for Enhanced Specificity

In addition to selective targeting, selective expression of effectors under spatially and temporally regulated promoters or regulatory elements provides another dimension to specific targeting.

Adenoviruses have been very effective gene delivery vectors, at least in vitro. They can accommodate about 3.5 kb of additional nucleotides in their genome, which is quite reasonable and sufficient to allow insertion of most commonly used gene expression cassettes. Many spatially and temporally regulated promoter driven expression cassettes were engineered into the AdV genome to express essential viral genes and other heterologous genes, including therapeutic proteins (interferons, monoclonal antibodies, cytokines, arresten, TNF-related apoptosis-inducing ligand (TRAIL) [156,157,158,159,160,161,162,163,164,165,166,167,168,169], and nucleic-acid-based effectors (RNAi-based regulatory elements, clustered regularly interspaced short palindromic repeats (CRISPR) and, transcription activator-like effector nucleases (TALENs).

#### 3.2.1. Protein-Based Effectors

Hemminki et al. used an OAdV to express granulocyte–macrophage colony-stimulating factor (GMCSF) under a viral E3 promoter (Ad5/3-D24-GMCSF). They were quite efficient in inducing a selective antitumor response. Hemminki et al. and others further demonstrated its effectiveness in patients who had failed conventional chemotherapy and radiation [156,157,158,159,160,170,171]. Similar to GMCSF, several other receptors and cytokines such as CD40L [162,163,164] and interferons [165,166,167,172,173,174] have also been evaluated. Yamamoto’s group has effectively used interferon expressing OAdVs to target pancreatic cancer in mice models and immunocompetent hamster models [172,173,174]. OAdVs have also been developed to express a monoclonal antibody against CTLA4. Hemminki’s group used a similar Ad5/3-D24aCTLA4 vector to express it in tumor cells and observed selective stimulation of T-cells in patients [168]. Most of these effectors are expressed with native adenovirus promoters, however using them with a specific promoter (temporally and/or spatially regulated promoter) may enhance the specificity.

#### 3.2.2. Nucleic-Acid-Based Effectors

Nucleic-acid-based effectors have significant advantages, as they do not need to express any proteins or undergo complicated post-translational modifications (as in the case of RNAi). Even when translation is necessary to produce functional protein–RNA complexes, they do not need to be produced in large quantities, as most of them are multiple turnover catalytic complexes (RNAi, CRISPRs, and TALENs) [175].

RNAi is an endogenous pathway of a cell or organism that defends against invasive genetic elements, such as viruses and transposons. RNAi-mediated pathways, which execute their functions through a multitude of small RNA-mediated pathways, including microRNAs, are key to maintaining cellular homeostasis and regulating metabolism [176,177,178,179]. Many miRNAs have been found to be involved in tumorigenesis by functioning as oncomiRs and tumor suppressors. Hence, they can also be potential targets for therapy. RNAi as a technique can be used to suppress mis-regulated oncogenes or oncomiRs via siRNAs, shRNAs, artificial miRNAs, anti-miRs, miRzips, sponges, ceRNAs, and artificial *lnc*RNAs [177,180,181,182,183]. However, their delivery in vivo has always been a concern [177,182,183,184]. Adenoviruses are promising in vivo delivery vectors, at least in some cases [185,186,187] and have the potential to be very useful in this regard.

Similar to many other viruses, AdVs also encode suppressors of RNAi, namely VA1 (viral associated RNA 1) and VA2 RNAs [188,189,190,191]. Usually removing such RNAi suppressors is known to enhance the efficiency of RNAi. Machitani et al. demonstrated this by efficiently suppressing a gene, but only with a non-replicating adenovirus [192]. On the other hand, the VA (viral associated) RNA is very important for oncolysis. VA RNA being a pro-viral RNA that is essential for efficient virus replication and inhibition of endogenous antiviral pathways, such as the PKR (Protein kinase RNA-activated) pathway, RNAi, and oligo adenylate synthase (OAS) mediated pathways is essential for efficient virus production and infectivity [193,194,195,196,197]. Removing VA-RNA leads to 20–60-fold decrease in AdV copies, which is very critical for effective oncolytic activity of adenoviruses [197]. Therefore, although the efficiency of the RNAi could be moderate, using replication of competent viruses without removing VA-RNA seems to be more effective for oncolysis [198,199,200,201]. Being very small in size, such RNAi-based effectors could be beneficial in designing combinatorial strategies where the size of the engineered recombinant virus genome is very critical to maintain efficient packaging [200,201,202].

As with genes, several endogenous miRNAs and other lncRNAs can be targeted to enhance the detargeting and retargeting ability of OAdVs [229]. For instance, liver, where most of the OAdV sequestration is observed, overexpresses miRNA122 and miR145. By incorporating multiple copies of miR122/145 target sites (in other words anti-miRs or sponges) in the 3′UTR of essential adenovirus open reading frames ORFs, significant reduction of AdV replication in liver was achieved [203]. Many other studies have deployed similar strategies by targeting miR122, miR145, miR148, miR21, and let7 [203,230,231,232,233,234,235,236,237]. These miRNAs, which are overexpressed in liver but not in tumors, are very useful in detargeting of AdV and they can be combined with other strategies. Since they are very small in size, it is a very feasible method.

Similar to genes, which can act as oncogenes or tumor suppressors, miRNAs can also promote (onco-miRs) or inhibit tumorigenesis (tumor suppressors). Typically, onco-miRs can be directly inhibited by using anti-miRs (sponges, ceRNAs) [204,205]. Ang et al. used OAdVs to target endogenous onco-miRs and inhibit EMT and tumor progression in triple-negative breast cancer (TNBC) [204]. They used an artificial lncRNA consisting of targets of nine onco-miRs overexpressed in TNBC cells, to suppress them simultaneously. In another study, an artificial lncRNA was used to suppress six different miRNAs simultaneously in sorafenib-resistant hepatocellular carcinomas (HCCs) [205]. These cancers are usually difficult to treat and this study showed the potential of RNAi-based therapies. These strategies are very versatile, as they can be used to target multiple genes or miRNAs together, which otherwise is very difficult to accomplish [238].

Sometimes, overexpression of miRNAs (tumor suppressor miRNAs) could be beneficial in reducing tumor burden by suppressing overexpressed oncogenes. For example, miR143 was overexpressed using OAdVs to suppress an oncogene, Kirsten rat sarcoma viral oncogene homolog (KRAS), in colorectal cancer cells [206] and miR199 was overexpressed to address HCC [203].

In absence of natural miRNAs to suppress oncogenes, artificial miRNAs, siRNAs, and shRNAs can be used [182,183,203,206,207]. The FGL2 gene in HCC tumors was targeted by using such artificial miRNAs to suppress angiogenesis [207].

Therefore, miRNAs, depending on their role, can be exploited for detargeting, retargeting, and as therapeutic agents by OAdVs. Combining them with other strategies will enhance the potency of OAdV-based therapies [123,124]. Luo et al. used a triple-regulated OAdV carrying miR143, survivin, and RGD to enhance the effects of OAdVs [124]. These RNAi methods are very versatile as they can be used to target multiple genes or miRNAs simultaneously, which is difficult using any other method. Since their size is relatively small, they can be easily engineered into some of the well-established and commonly used vectors [200,201,202].

Unlike RNAi, which is mostly a post-transcriptional gene silencing mechanism, TALENs and CRISPRs have revolutionized genome engineering and have opened up new avenues of targeted therapy [175,239,240,241]. Oncogenes and tumor suppressors can be manipulated using these techniques [241,242]. Again, their delivery has been a big challenge. In some cases, adenoviruses were effectively used to deliver these programmable nucleases or modifiers [243,244,245,246,247]. Among these genome engineering techniques, the nucleotide components determining the sequence specificity in CRISPRs (guides) are smaller than those of TALENs. Hence, the CRISPR-based strategies seem more promising than TALENs. They can potentially be accommodated in a non-helper-dependent viral vector, although most of the studies so far have used helper-dependent viruses [175,246,248,249,250,251,252]. Maggio et al. delivered functional gRNAs and human-codon-optimized Cas-9 into a diverse array of human cells [244]. Although some gene editing-based studies have shown promising results and are going through clinical trials [253,254], creating mutations in the genome may not be a safe strategy, due to its permanence and potential off-target activities. This may impart undesirable and irreversible damages to genome. However, other CRIPR based tools like, modified CRISPR-based base editors, suppressors and enhancers (which do not cleave the target) could be safer alternatives [248,249,250,255,256,257,258].

### 3.3. Selective (Conditional) Replication of Viruses in Specific Cells

The ability of oncolytic adenoviruses to replicate specifically in cancer cells will provide them a significant advantage over traditional chemotherapies. In order to achieve this, an oncolytic adenovirus must: (i) demonstrate stability in vivo and avoid potential degradation or uptake by non-target cells; and (ii) preferentially infect and replicate in cancer cells, thereby providing limited delivery to normal cells via a conditionally replicative adenovirus (CRAd) [259]. There are mainly two types of CRAds: one uses a tumor-specific promoter to induce replication and the second uses the interaction of essential viral genes with tumor-specific proteins.

Several genes are uniquely expressed or significantly overexpressed in tumors, often due to their promoters being active in those tumor cells and microenvironments. Promoter-based CRAds are designed to express essential viral genes with such promoters so that virus replication is possible only in those cells. The most prominent examples include Cox2 in gastric cancer and pancreatic cancer [69,209,210,211] and survivin in adult T cell leukemia or lymphoma [212,213,214,215] and lung, ovarian, and pancreatic cancers. The HIF-responsive promoter in gliomas and meduloblastomas [216,217]; and hTERT (human telomerase reverse transcriptase) in bone and soft tissue sarcomas, as well as prostate, ovarian, esophageal, and GI cancers [218,219,220,221,222,260,261], among many others.

The second type of CRAds involve mutations or deletions in the E1 region to allow tumor-specific replication [223,262]. The E1 region of the adenovirus is involved in its replication and consist of 2 genes, E1A and E1B.These two genes encode proteins essential for a “productive” adenovirus replication cycle [263]. E1A encodes for proteins 243 R and 289 R, which induce transcription of early viral gene regions and stimulate the entry of infected cells to the S phase of the cell cycle. E1B encodes for proteins E1B19K and E1B55K, which are involved in inhibition of apoptosis via their interaction with p53 and Rb proteins in infected cells, among other important functions [263]. E1A causes the induction of the S phase, which has an apoptotic response and must be inhibited by E1B gene products in order to facilitate successful viral replication cycle [264]. Mutations or deletions in the E1 region abolish the interaction of viral proteins with either p53 via E1B [265,266] or pRb via E1A [267,268] to target tumor cells defective for those gene products. ONYX-015 is a OAdV that lacks the EB1B region and selectively replicates in mutated p53 tumors [224]. Similarly, Ad∆24 is a OAd that has a mutation in E1A and restricts replication to retinoblastoma protein (pRb) mutated cancer cells [225]. Although these CRAds are better than first-generation CRAds, such as onyx-015, they were not very effective in clinical trials when used alone [269]. To target cancer cells that harbor activating mutant KRAS (KRAS^aMut^) but spare p53^wild^ normal cells, Liu et al. [270] constructed the Δ*p53RE*P2 promoter with deletion of its p53-response elements. This was used to regulate the expression of the hdm2 transgene in a novel E1B-55kD-deleted CRAd, the Ad-KRhdm2. The virus showed selective replication in colorectal cancer cells with KRAS mutation and P53 WT. Furthermore, Kim et al., in an effort to augment radiation therapy, generated double E1B 19kDa and E1B 55kDa deleted oncolytic adenovirus (Ad−ΔE1B55), which when combined with radiation therapy showed greater cytotoxicity than the single E1B-55kDA-deleted oncolytic AdV [271]. Similarly, Yoon et al. [272] demonstrated that combined deletion of E1B 19kDA and E1B 55kDA increased the cytotoxicity when combined with cisplatin, which is a standard of care for many cancers, such as ovarian, cervical, breast, and bladder cancers.

### 3.4. Combinations of the Abovementioned Strategies and Other Anticancer Treatments for Enhanced Specificity and More Effective Therapeutic Effect

We believe a combination of detargeting and retargeting strategies is more effective than either of them alone. This has been demonstrated by combinations of targeted modifications in the host-interacting domains of viral proteins, such as HVR regions of hexon and AB-loop regions of fiber [55,78,100].

Since a replication-competent adenovirus genome can accommodate up to ~3.5 kb of extra genetic material into its capsid, other strategies to increase the specificity and efficiency can be combined. Selective expression of effectors and conditional replication-based strategies are best combined with detargeting- and retargeting-based vector designs.

Nucleic-acid-based effectors (RNAi-based) are smaller in size and relatively easier to combine with other strategies. Therefore, miRNAs, depending on their role, can be exploited for detargeting, retargeting and also as therapeutic agents by OAdVs [202,208]. Luo et al. used a triple-regulated OAdV carrying miR143, survivin, and RGD to enhance the effects of OAdVs [200,202]. Lou et al. overexpressed miR34a combined with overexpression of interleukin-24 (IL-24) using hTERT promoter-driven E1A-D24-type CRAds to test against HCC [208]. These methods are very versatile, as they can be used to target multiple genes or miRNAs simultaneously, which is difficult to do using any other method. Some studies have targeted up to nine miRNAs at once [205]. Since their size is small they can be easily engineered into well established and commonly used vectors [200].

Protein-based effectors such as interferon-α show toxicity when administered systemically [273]. Yamamoto’s group developed an OAdV (Ad5/Ad3-Cox2-ΔE3-ADP-IFN) that combines multiple strategies, including conditional replication, detargeting and retargeting via the RGD motif and Ad5/3 chimeric fiber, overexpression of ADP and expression of interferon-α to target pancreatic cancer [173,174]. Another OAdV (CRAd-arresten-TRAIL) was developed to combine conditional replication, engineered fibers, and expression of two protein effectors, arresten and TRAIL [169]. Hemmiki’s group used OAdVs (Ad5/3-Δ24aCTLA4) to express checkpoint inhibitors such as anti-CTLA4 in combination with *Rb*/p16-dependent CRAd and chimeric fiber [167] to observe enhanced stimulation of T-cells in patients with advanced cancer.

Combining these strategies to improve tumor selectivity and specificity with other anticancer treatments, such as chemotherapy and radiotherapy, can further improve the therapeutic outcomes [274]. An interferon-expressing OAdV used in combination with chemotherapy and radiotherapy significantly reduced the systemic toxicity and increased antitumor effects [172]. OAdVs seem to make targeted cells more sensitive to radiation-induced damages by suppressing dsDNA break repair pathways [226,275,276,277]. Their interaction with other conventional chemotherapies are not clear but they have been found to be effective, as shown in the case of onyx-015 and other interferon-expressing CRAds [172,226,227]. In some cases, specific inhibitors of oncogenes were combined with CRAds. Nutins, known inhibitors of MDM2, which in turn suppresses p53, were used in combination with a CRAd designed to overexpress p53 to get a much better effect than with p53-overexpressing AdV alone [228].

Overall, combinations of these strategies enhance tumor selectivity and specificity. Depending on the context, such as the type of tumor, genes needed to be targeted, and other conventional therapies available, one can tailor multiple combinatorial strategies for a better therapeutic outcome.

## 4. Conclusions

Oncolytic viruses are novel and effective tools for delivering targeted therapy to cancer cells. They have the potential to target any gene that is specifically expressed or overexpressed on the surface of a cancer cell. Most importantly, OVs can exploit genes which are overexpressed, irrespective of their functional relevance to tumor biology. This makes a large number of genes targetable which could not be targeted earlier by conventional therapies. The flexibility of DNA manipulation to drive detargeting and retargeting of these viruses, combined with conditional replication and targeted expression, allows for the combination of multiple good tools into one. This may enable us to design a therapy that is more specific, targeted, and effective. Furthermore, the combination of these new therapies with conventional anticancer therapies such as radiation and chemotherapy is very promising and may provide additional benefits to cancer patients.

## Figures and Tables

**Table 1:** Detargeting and retargeting strategies with representative studies.

Strategy	Examples	References
***a. Selective Detargeting And Retargeting Towards Specific Receptors/Cells.***		
**Detargeting of AdV structural proteins**		
**Detargeting in Hexon**		
Insertion of lysine in hexon	Liver detargeting	[60]
Insertion of targeting peptides	Skeletal muscle cells, liver detargeting	[64,65,76,77,78,79]
Insertion of large universal acceptor proteins (BAP)	Liver detargeting	[80]
HVR swap	Liver detargeting, breast cancer, immune evasion	[57,59,61]
Drugs (warfarin, Snake venom protein X-Bp)	Liver detargeting	[62,63]
Hexon and pIX	Liver detargeting, HCC, ovarian carcinoma, melanoma	[81,82,83]
**Detargeting of Fiber**		
Modified HI-loop Modified AB-loop Insertion of proteins or universal acceptors	neuronal cells, brain endothelial cells, vascular endothelial cells, prostate cancer, lung cancer, pancreatic cancer, muscle cells, colorectal cancer	[66,67,85,86,87,88,89,90,91,92,93,94,95,96,97] [55,100] [87,88,98,99,101,102,103,104,105,106,107,108,109,110,111,112,113,114]
**Infectivity Selective Screening for Specific Targeting: Strategies and Importance**		
Phage display and other screening: Insertion of selected peptides into AdV proteins	Liver detargeting, neuronal cells, brain endothelial cells, vascular endothelial cells, prostate cancer, lung cancer, pancreatic cancer, muscle cells, colorectal cancer	[79,121]
Adenovirus-library-based screening: Direct modification of AdV libraries.	Liver detargeting, pancreatic cancer, colorectal cancer.	[55,100]
**Retargeting of AdV by Affinity Modifiers**		
Antibody–antibody-based bridging molecules	Anti-AdV protein conjugated to VEGFR, TIE-2, integrins, EPCAM, EGFR, HER2, Endoglin, HMWMAA	[127,128,129,130,131,132]
Antibody–ligand-based bridging molecules	anti-AdV protein conjugated to Folate, TNFa, IGF1, EGF	[133,134]
Peptide–antibody-based bridging molecules	p75 neurotropin receptor on hepatic stellate cells	[135]
Soluble receptors conjugated to ligands (sCAR, FX)	EGF, anti-Cd40 ScFv, ApoE ligand, anti-ErbB2, anti-CEA, Polysialyc-acid (PSA), CXCL12	[136,137,138,139,140,141,142,143,144,145]
BiTE, Leucine-zipper-based linkers	CD44v6, anti-B-cell maturation antigen, FR-α, EGFR, EpCAM, carcinoembryonic antigen, CD40	[147,148,149,150,151,152,153,154]
***b. Selective Expression of Effectors (Inhibitors/Enhancers) in Specific Cells.***		
Protein-based effectors	Interferons, GM-CSF, IL12, CD40L, CTLA4	[156,157,158,159,160,161,162,163,164,165,166,167,168,170]
Nucleic-acid-based effectors.	Liver detargeting, miR122, miR145, miR148, miR21, let7, KRAS, breast cancer, Hepatocellular carcinoma, colorectal cancer cells	[202,203,204,205,206,207,208]
***c. Selective replication of virus in specific cells.***		
Promoter-based CRAd	Lung, prostate, ovarian, pancreatic cancer, adult T cell Leukemia/Lymphoma, glioma, meduloblastoma, sarcomas	[69,209,210,211,212,213,214,215,216,217,218,219,220,221,222]
CRAds based on Interaction of essential viral genes withTumor-specific proteins	colon, breast, non-small cell lung, head and neck, and pancreatic tumors, cervical carcinoma, glioblastoma, cancers with disturbed Rb pathway	[172,223,224,225,226,227,228]
